# Profiling the Paralytic Effects and Lethality of Cone Snail Venom Toxins Using Nanofractionation Analytics with *In Vivo* Zebrafish Larvae Assays

**DOI:** 10.3390/toxins17100504

**Published:** 2025-10-13

**Authors:** Jeroen Kool, Arif Arrahman, Haifeng Xu, Jiaxing Liu, Richard J. Lewis, Christian Tudorache, Fernanda C. Cardoso

**Affiliations:** 1 Amsterdam Institute of Molecular and Life Sciences, Division of BioAnalytical Chemistry, Department of Chemistry and Pharmaceutical Sciences, Faculty of Science, Vrije Universiteit Amsterdam, De Boelelaan 1085, 1081 HV Amsterdam, The Netherlands; 2 Centre for Analytical Sciences Amsterdam (CASA), 1081 HZ Amsterdam, The Netherlands; 3 Institute for Molecular Bioscience, The University of Queensland, Brisbane, QLD 4072, Australia; 4 Institute Biology Leiden, Leiden University, 2333 BE Leiden, The Netherlands

**Keywords:** cone snail venom, nanofractionation analytics, high throughput zebrafish embryo screening, paralysis, mass spectrometry

## Abstract

This study presents nanofractionation analytics coupled with *in vivo* profiling of zebrafish embryo paralysis and lethality in response to toxins in cone snail venoms. The focus of this study is on the development of this approach using venoms of *Conus marmoreus*, *Conus ebraeus*, and *Conus bandanus*. In brief, cone snail venoms were separated using reversed-phase chromatography following high-resolution nanofractionation on microplates with parallel mass spectrometry, enabled via a post-column flow split. All collected fractions were dried overnight, followed by assays on zebrafish embryos. For the paralysis assessment, we monitored swimming behavior and swimming distance and found that exposure to cone snail toxins led to paralysis and decreased movement and swim distance. To correlate the masses of eluted toxins with their paralyzing effects and potency, we compared the fractionation retention time versus normalized swimming distance. This allowed identification of the masses of toxins with paralyzing bioactivity, which were predominantly conopeptides. To assess lethality, zebrafish embryos were exposed to fractionated toxins for 24 h, after which they were inspected. The lethal doses and correlated toxins were identified by comparing retention times of fractionation versus the lethal dose values calculated for each fraction. We found that the most lethal venom was from *C. bandanus*, displaying the largest number of lethal peptides, followed by *C. marmoreus* and *C. ebraeus*. On the other hand, the most paralytic venom was from *C. ebraeus*, presenting a higher number of peptides with non-lethal paralytic effects, followed by *C. bandanus* and *C. marmoreus*. This study provides a pipeline to rapidly identify paralytic and lethal cone snail venom toxins using the zebrafish embryo model.

## 1. Introduction

Cone snails are amongst the most extraordinary clade of venomous animals. Their venoms comprise a complex mixture of cysteine knot peptides that target ion channels and receptors to induce harmful effects, including death. These toxins or conopeptides assist with prey capture and defense [1]. Despite such harmful effects, research into cone snail venoms has revealed their potential as pharmacological tools and novel drugs to treat complex neurological disorders [2,3]. This is evident from the discovery of the analgesic drug ziconotide derived from the venom of *Conus magnus* and approved to treat neuropathic pain [4,5]. Ziconotide is a selective inhibitor of the voltage-gated N-type calcium channel involved in pain transmission and abrogates pathological synaptic activity on sensory neurons. This discovery opens up new opportunities for conopeptide-based drugs that target a wide range of ion channels and receptors involved in disease pathophysiology [6,7,8].

Modern discovery pipelines using integrative approaches accelerate the identification of crude venoms and venom components with applications in fundamental research and drug development. High-throughput (HT) functional assays are imperative in this process for robust pharmacological characterization of bioactivities and molecular targets modulated by venoms [9]. These assays, often colorimetric or fluorescence-based *in vitro* assays, use enzymatic assays or cellular assays to generate bioactivity information and define molecular pharmacology. Another approach harnesses HT *in vivo* assays using small animals such as *Caenorhabditis elegans* and *Danio rario* (zebrafish), to measure bioactivities, and often uses behavioral measurements to determine induced biological effects [10,11]. Combined, HT functional *in vitro* and *in vivo* assays deliver a comprehensive overview of the molecular targets and bioactivities of venoms and their components [12].

Venomic studies determine protein and peptide sequences and identify those that are complementary to functional screens [13,14]. Proteomic analyses using mass spectrometry of crude venoms and fractions provide identification of the molecular mass and partial residual sequence of venom components, facilitating the correlation of toxins, bioactivity, and, in some instances, molecular targets based on other toxins in previously characterized venom components. Limitations in this approach are overcome by transcriptomic analysis that produces complete open reading frames for these toxins correlated with partial sequences and masses determined by proteomics. Combined, proteomics and transcriptomics allow for the characterization of the venom components at the molecular level and complement studies of bioactive venom components when applied in conjunction with HT bioassays.

In this study, we developed a new pipeline for the characterization of cone snail venom components and their associated bioactivities by combining three elements: (i) high-resolution nanofractionation; (ii) mass-spectrometry assessment; and (iii) *in vivo* zebrafish assays. Zebrafish assays measure the paralytic effects and lethality of toxins. We demonstrate the feasibility of this approach by investigating venoms from three species of cone snail, *Conus marmoreus*, *Conus ebraeus*, and *Conus bandanus*. Our results showed that the venoms of these species are rich in paralytic and lethal peptides, as demonstrated by the zebrafish bioassays and mass spectrometry results. The most lethal venom was from *C. bandanus*, displaying a larger number of lethal peptides, followed by *C. marmoreus* and *C. ebraeus*. On the other hand, the most paralytic venom was from *C. ebraeus*, presenting a higher number of peptides with non-lethal paralytic effects, followed by *C. bandanus* and *C. marmoreus*. This proof-of-concept study demonstrates the feasibility of rapid profiling of venoms using nanofractionation analytics incorporating mass spectrometry and *in vivo* assays to identify bioactive components in cone snail venoms associated with paralytic and lethal effects *in vivo*.

## 2. Results and Discussion

This study presents a pipeline for rapidly assessing cone snail venoms for the presence of toxins with *in vivo* paralyzing properties and/or lethality in zebrafish embryos. The focus is on demonstrating the analytical workflow for which the venoms of *Conus marmoreus*, *Conus ebraeus*, and *Conus bandanus* were evaluated. In the following three sections, the results for each of these three cone snail venoms are presented in individual figures. Mass spectrometry (MS) data and *in vivo* bioassay data chromatograms were superimposed to correlate bioactivity with cone snail toxin accurate masses plotted as eXtracted Ion Chromatograms (XICs) from the Total Ion Chromatogram (TIC). The top chromatogram in each figure is the TIC, which is followed by the Lethal Dose 50 (LD_50_) and the paralysis chromatograms, and then followed by three windows with XICs of *m/z*-values of ions of cone snail toxins representing their accurate masses. The accurate masses of these toxins are given in the side panels and were calculated from their *m/z*-values. The accurate masses of toxins correlating with paralysis and/or an LD_50_ are described in the upper TIC.

When more than one toxin was tentatively matched to an *in vivo* bioactivity, all accurate masses of toxin candidates are given as a list, in which the most likely candidate is given in bold. As actual concentrations of the fractionated cone snail toxins cannot be measured using the low amounts of venom fractionated, bioactivity potencies are empirically presented. For this, the LD_50_ evaluation presents lethality as negative peaks in which the more potent and lethal the toxin is, the larger the negative peak observed. For the paralysis evaluation, the paralyzing ability of fractionated cone snail toxins is represented as negative peaks with no paralyzing effects shown as the average normal distance swum baseline (i.e., 1 on the y-axis) and full paralysis as zero distance swum (i.e., 0 on the y-axis). This qualitative bioassay can therefore be used to estimate the potency of toxins for the two *in vivo* bioassays. The XICs were plotted in three graphs to facilitate data visualization and analysis, considering the large amount of relevant data to interpret. For *Conus marmoreus*, the XIC graphs were plotted based on XIC intensity range since the relevant toxins displayed a wide range of intensities (i.e., 10^5^ to 10^7^). For *Conus ebraeus* and *Conus bandanus*, all relevant toxins had XIC intensities in the 10^6^ range. Therefore, the XICs in these cases were plotted in the XIC graphs based on their retention time frame (i.e., 14–16 min, 16–18 min and 18–21 min).

### 2.1. Conus Marmoreus

**Figure 1:**
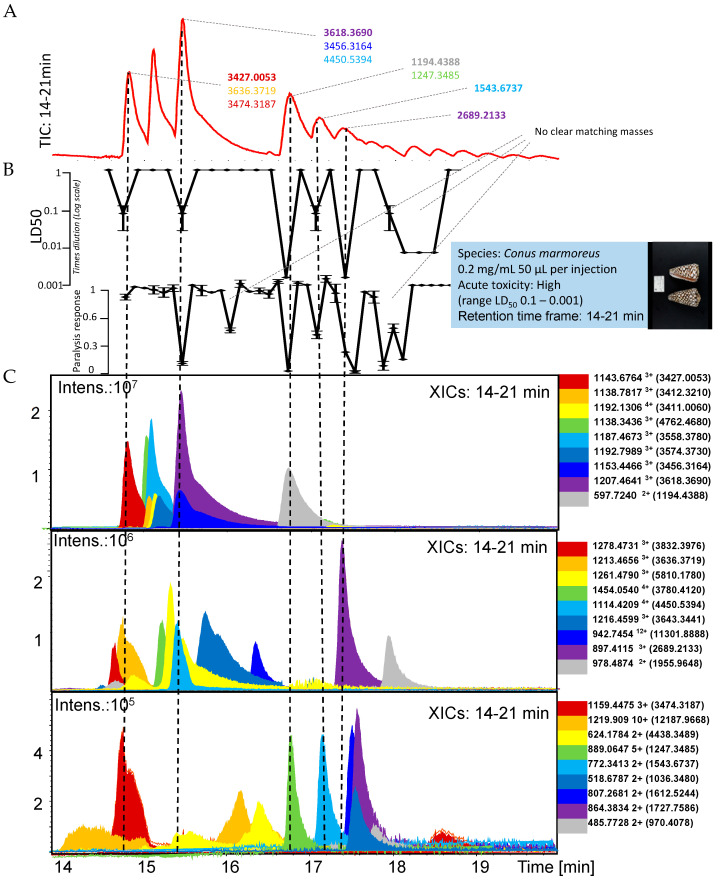
**Integrated chromatographic results of the analysis of *Conus marmoreus* venom**. (**A**) Upper graph shows the Total Ion Chromatogram (TIC) of the retention time window where the cone snail toxins eluted (i.e., 14 to 21 min). Tentative accurate masses of toxins corresponding with *in vivo* bioactivity peaks (shown in the next two chromatographic bioactivity representations in (**B**)), which were correlated in retention time and peak shape to one or more of the lower three eXtracted Ion Chromatograms (XICs in (**C**)), are also given here. The second and third chromatographic representations from above show the LD_50_ and paralysis data, respectively. The lower three chromatographic graphs show the XICs, sorted on intensity range (i.e., 10^7^ as the first XIC graph; 10^6^ as the middle XIC graph; and 10^5^ as the lower XIC graph).

The *Conus marmoreus* venom findings are represented in Figure 1. We identified six fractions representing clear lethality and six fractions representing paralysis, of which the last bioactivity peak was observed in three consecutive fractions. The fractions eluted at 15.3 min, 16.7 min, 17 and 17.3 min showed both paralysis in the 50 min paralysis bioassay and lethality in the 24 h incubation LD_50_ bioassay. The most likely candidate toxins corresponding to 15.3, 16.7, 17.2 and 17.4 min peaks had accurate masses of 3618.3690, 1194.4388, 1543.6737 and 2689.2133, respectively. A toxin eluting at 14.7 min showed lethality, but no significant paralytic effects. The tentative toxin accurate mass assigned to this bioactivity was 3427.0053. In the paralysis assay, we observed a bioactivity peak at a retention time of around 16 min, for which no clear matching XIC was found. In addition, this toxin did not show a lethal response. It is possible that this paralysis-causing toxin was metabolized and/or degraded or oxidized rapidly, thereby not causing lethality after 24 h exposure.

The broad peaks observed after 17.7 min in both paralysis and lethality bioassays could not clearly be correlated to any eluting toxin from the XIC data due to their broad peak shape (for the LD50 response) and split peak behavior (for the paralysis bioassay). Such peak shapes were not observed for any toxin when inspecting the MS data (by plotting XICs). Possibly, these bioactivities were caused by several co-eluting cone snail toxins, with a predominant toxin with an accurate mass of 1955.9648.

### 2.2. Conus Ebraeus

**Figure 2:**
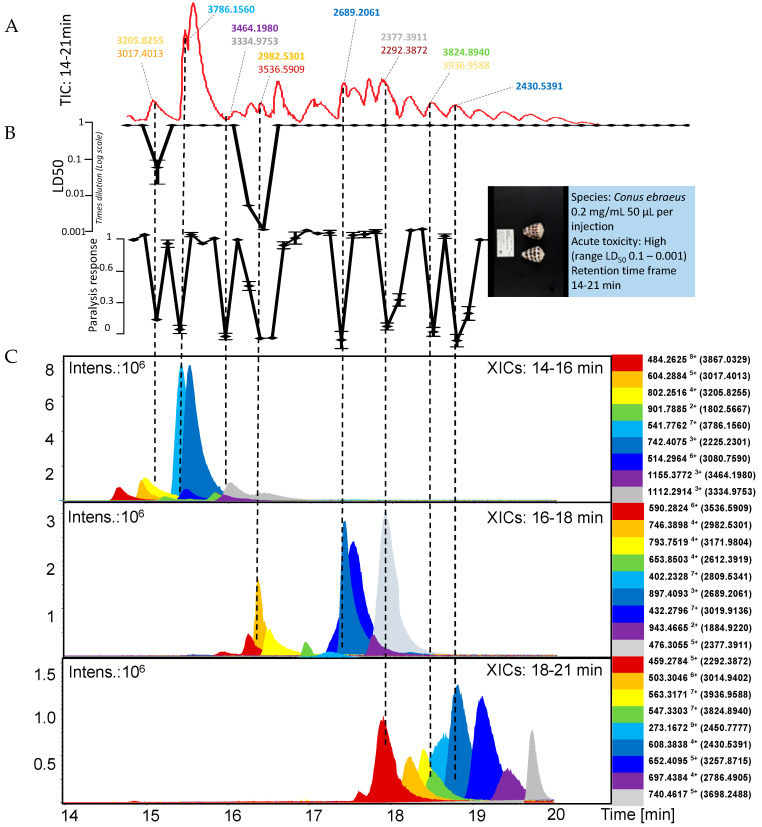
**Integrated chromatographic results of the analysis of *Conus ebraeus* venom**. (**A**) The upper graph shows the Total Ion Chromatogram (TIC) of the retention time window where the cone snail toxins eluted (i.e., 14 to 21 min). Tentative accurate masses of toxins corresponding with *in vivo* bioactivity peaks (shown in the next two chromatographic bioactivity representations in (**B**)), which were correlated in retention time and peak shape to one or more of the lower three eXtracted Ion Chromatograms (XICs in (**C**)), are also given here. The second and third chromatographic representations from above show the LD_50_ and paralysis data, respectively. The lower three chromatographic graphs show the XICs, sorted on retention time frame (i.e., 14–16 min as the first XIC graph; 16–18 min as the middle XIC graph; and 18–21 min as the lower XIC graph).

From Figure 2, the *Conus ebraeus* venom produced eight peaks showing a clear paralysis response, from which two also showed lethality. These two eluted at 15.0 and 16.3 min, for which tentative accurate masses of the toxins were 3205.8255 and 2982.5301, respectively. The toxins that caused paralysis and were not lethal to the zebrafish embryos were eluted at 15.3 min, 15.9 min, 17.4 min, 17.9 min, 18.5 min and 18.8 min and had corresponding most likely tentative accurate masses of 3786.1560, 3464.1980, 2689.2061, 2377.3911, 3824.8940 and 2430.5391.

### 2.3. Conus Bandanus

**Figure 3:**
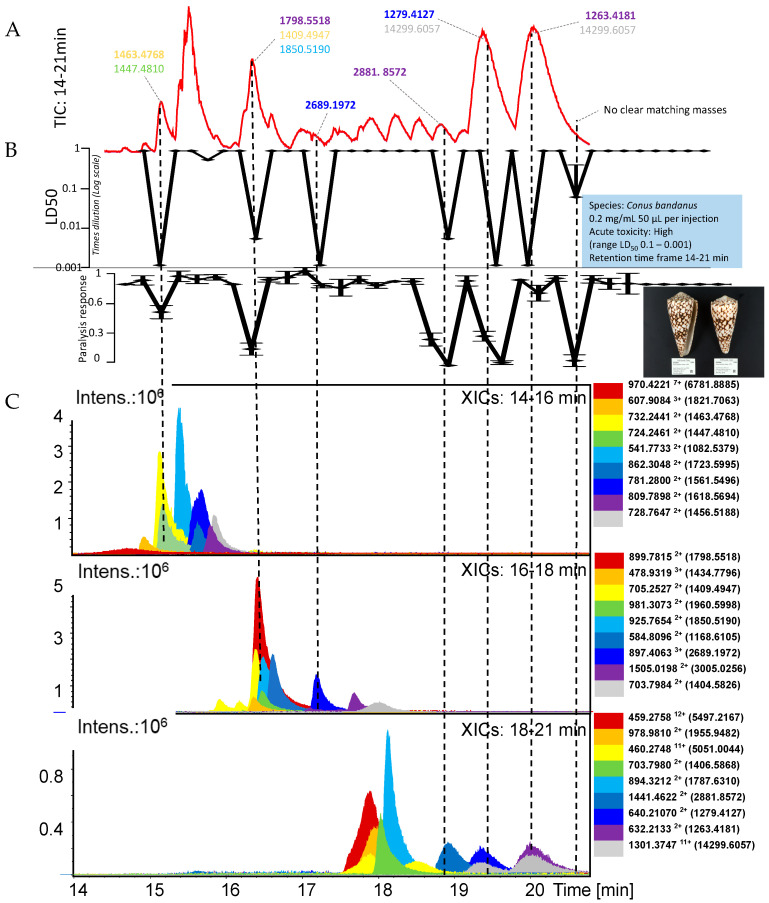
**Integrated chromatographic results of the analysis of *Conus bandanus* venom**. (**A**) Upper graph shows the Total Ion Chromatogram (TIC) of the retention time window where the cone snail toxins eluted (i.e., 14 to 21 min). Tentative accurate masses of toxins corresponding with *in vivo* bioactivity peaks (shown in the next two chromatographic bioactivity representations in (**B**)), which were correlated in retention time and peak shape to one or more of the lower three eXtracted Ion Chromatograms (XICs in (**C**)), are also given here. The second and third chromatographic representations from above show the LD_50_ and paralysis data, respectively. The lower three chromatographic graphs show the XICs, sorted on retention time frame (i.e., 14–16 min as the first XIC graph; 16–18 min as the middle XICs graph; and 18–21 min as the lower XICs graph).

In Figure 3, the *Conus bandanus* venom produced five clear peaks showing both paralysis and lethality responses. The toxin accurate masses tentatively assigned to these bioactivities eluted at 15.0 min, 16.3 min, 18.8 min, 19.3 min and 20.6 min. Their most likely accurate masses retrieved were 1463.4768, 1798.5518, 2881.8572 and 1279.4127 for the first five eluting peaks. For the last peak (20.6 min), no XIC trace could be found that clearly matched, and as such, no tentative accurate mass could be assigned to this bioactivity peak. The LD_50_ data also showed two peaks (eluting at 17.1 min and 19.9 min) with lethal response in the zebrafish embryos, which was not related to paralysis. The tentative toxin accurate masses assigned to these two bioactivities were 2689.1982 and 1263.4181.

It is important to note that not all peptides detected in our MS analyses were associated with measurable biological activity in the zebrafish assays. For example, MiXXVIIA (accurate mass: 3412.3210 Da) was detected in a fraction that did not induce either paralysis or lethality under our assay conditions, whereas conotoxin-GS (3618.3690 Da) was clearly correlated with strong paralytic and lethal responses. Likewise, several other peptides were present in fractions that showed no bioactivity. This observation highlights that detection of a peptide in the venom does not necessarily imply acute activity in the zebrafish model, which may be due to limited abundance, degradation, or distinct pharmacological profiles that are not captured in the current *in vivo* assays. For transparency, we therefore explicitly point out that our study identifies both bioactive toxins and peptides without detectable activity in the bioassays we used.”

We finally investigated which of the accurate masses of the conotoxins that we have described in our study are also given in the Conoserver.org database (a database of toxins isolated from Cone snails), and for those found, if there are studies describing certain biological activities of these toxins. From this investigation, we constructed a table (given in the Supplementary Materials as ‘SI_Conoserver_org_search_results.xlsx’) that lists cone snail venom toxins found in our study that induced paralysis and/or lethality in zebrafish embryos and have published bioactivity data retrieved from the Conoserver database [15,16,17,18,19,20,21,22,23,24,25,26,27,28,29,30,31,32,33,34,35,36,37,38,39,40,41,42,43,44,45,46,47,48,49,50,51,52,53,54,55,56,57,58,59]. The table includes our accurate masses found, the theoretical mass retrieved from the Conoserver database, as well as conotoxin names, species of origin, conotoxin classification, and reported molecular targets or pharmacological effects (including literature on conotoxins not dealing with biological activities).

This paragraph deals with the results obtained from the cone snail *Conus marmoreus* investigated in relation to the data available in the Conoserver.org database. Several cone snail toxins exhibited both paralysis and/or lethality in zebrafish embryos and were also previously described in the literature with relevant bioactivities, providing insight into their likely mechanisms of action. One such toxin is MiXXVIIA (accurate mass: 3412.3210 Da) from *Conus miles*, a G2-superfamily conotoxin reported to have anti-apoptotic and proliferative activity [48]. While not directly neurotoxic, such modulatory effects on cell survival pathways could contribute to systemic toxicity. Another toxin, Ml6.6 (3411.0060 Da) from *Conus miliaris*, has documented activity on human sodium channels [49]. Given the central role of these channels in neuronal and muscular excitation, their disruption can explain the paralysis and death seen in exposed embryos. This suggests a direct mechanism for the neurotoxic effects observed. The toxin conotoxin-GS (3618.369 Da), from the highly venomous *Conus geographus*, is a sodium channel inhibitor [50]. Sodium channels are crucial for neuronal signal transmission, and their inhibition leads to rapid paralysis, a hallmark of this species’ venom effect on prey (and humans). This supports a clear mechanistic correlation between the detected toxin and the lethal or paralytic effects observed in zebrafish embryos.

This paragraph deals with the results obtained from the cone snail *Conus ebraeus* investigated in relation to the data available in the Conoserver.org database. Toxin Vx11.1 (3786.1560 Da) from *Conus vexillum* inhibits several ion channels, including Kv1.1, Kv1.2, Kv1.3, HERG, Kir2.1, and Nav1.5 [51]. These channels are essential for neuronal, muscular, and cardiac excitability. Their inhibition provides a clear mechanistic basis for the paralysis and/or death observed in zebrafish embryos. Conotoxin Vi6.4 (3080.7590 Da) from *Conus virgo* is reported as a neuroactive peptide with modulatory effects [52]. Although its precise molecular targets are not defined, such central nervous system activity may plausibly contribute to the paralysis and/or lethality observed in zebrafish embryos. Conotoxin C074 (2982.5301) from *Conus catus* is reported to modulate NaV channels, neuronal nicotinic acetylcholine receptors, and CaV2.2 channels [53]. These ion channels are essential for neuromuscular signaling, and their disruption aligns well with the observed results in the zebrafish embryos. For toxin Tr6.2 (3171.9804 Da) from *Conus terebra* is reported as a neuroactive peptide with modulatory effects [53]. Although its precise molecular targets are not defined, such central nervous system activity probably contributes to zebrafish paralysis/lethality. Conotoxin Ac6.4 (2612.3919 Da) from *Conus achatinus* is reported as a CaV channel blocker [54]. Voltage-gated calcium channels are essential for neurotransmitter release and muscle contraction, and their inhibition provides a clear mechanistic basis for paralysis and/or lethality observed in zebrafish embryos. Conotoxin KIIIA (1884.9220 Da) from *Conus kinoshitai* has been shown to inhibit human voltage-gated sodium channels, which are critical for nerve and muscle excitability [55]. This mode of action aligns well with the paralytic and lethal effects observed in zebrafish embryos exposed to this venom component.

This paragraph deals with the results obtained from the cone snail *Conus bandanus* investigated in relation to the data available in the Conoserver.org database. The accurate mass found of 1618.5694 Da matches in the Conoserver with two conotoxins for which literature studies were found dealing with bioactivity assessment [56]. The first one is α-conotoxin CIA from *Conus catus* that targets both muscle-type nicotinic acetylcholine receptors and the neuronal α3β2 subtype with high affinity. This dual action on key components of neuromuscular transmission offers a likely explanation for the paralytic and lethal effects observed in zebrafish embryos. The second one is conotoxin SrVA from *Conus spurius*, for which it has been reported to exhibit bioactivity at the central nervous system [57]. While its precise molecular targets are not specified, its CNS activity suggests a potential role in the paralysis and/or lethality in zebrafish embryos. With an accurate mass found of 1456.5188 Da, there was a match found in the Conoserver with conotoxin SIA from *Conus striatus*, for which it has been reported that it displays moderate neurotoxic activity in mice by blocking muscle-type nicotinic acetylcholine receptors [44]. This interference with neuromuscular signaling likely underlies the bioactivity observed in the zebrafish embryos. Conotoxin MrIA (1409.4947 Da) from *Conus marmoreus* is a selective inhibitor of human norepinephrine transporters (hNET) [59]. By interfering with neurotransmitter reuptake, this peptide may contribute to neurological dysfunction that aligns with the results from the zebrafish embryos. Acting on nicotinic acetylcholine receptors, conotoxin AnIB (1787.6310 Da) from *Conus anemone* may disrupt normal neuromuscular signaling [60]. Such interference offers a likely explanation for the paralysis and/or lethality observed in zebrafish embryos.

A limitation of the present approach is that the correlation of detected peptide masses with *in vivo* effects relies on co-elution in nanofractionation, which may still contain multiple peptides per fraction. This makes it challenging to assign bioactivity unambiguously to a single compound without further purification or structural confirmation. Moreover, some peptides were detected in fractions that did not produce measurable paralysis or lethality in zebrafish, indicating that not all venom components are active in this particular *in vivo* model, or that their activity is outside the sensitivity window of our assay conditions. Another limitation is that the zebrafish embryo model, while highly valuable for rapid *in vivo* screening, cannot fully capture all aspects of mammalian or human physiology, and thus may not predict therapeutic windows or off-target effects in higher organisms. Despite these limitations, the combination of nanofractionation, parallel MS, and zebrafish bioassays provides a powerful high-throughput pipeline to rapidly identify candidate bioactive peptides from complex venoms. Future perspectives include integrating this workflow with advanced structural characterization (e.g., top-down proteomics or other venomics strategies) and target deconvolution strategies (e.g., electrophysiology or receptor binding assays) to confirm the mode of action of identified peptides. In addition, coupling this platform to transcriptomic databases and automated fraction annotation will further accelerate the discovery of novel conopeptides with biomedical potential.

### 2.4. Concluding Remarks

In this study, we combined nanofractionation analytics and *in vivo* zebrafish embryo assays to rapidly determine cone snail venom components that induce paralysis and/or death in zebrafish. We used reversed-phase chromatography connected to electrospray ionization mass spectrometry and parallel behavioral and lethal bioassaying in zebrafish to determine bioactive venom components and identify their molecular masses. From this study, we determined that the most potent lethal venom components were found in *C. bandanus* venom, followed by *C. marmoreous* and *C. ebraeus* venoms. On the other hand, the most paralytic venom components were from *C. ebraeus*, followed by *C. bandanus* and *C. marmoreus*. These venoms are known to comprise peptides called conopeptides, which are often cysteine-rich, with diverse functions and classified into families based on their precursor sequences and targets. These targets include acetylcholine receptors, GABA receptors, adrenoreceptors, noradrenaline, and sodium, calcium and potassium channels regulating physiology in the neuronal, cardiovascular and muscle-skeletal systems that support the *in vivo* results obtained in this work [61,62,63,64,65,66]. Profiling the venom composition and bioactivities using high-throughput platforms is imperative to advance research in the fields of venom evolution. It also facilitates the identification of novel bioactive entities capable of influencing physiological functions, which have the potential to become new biopharmaceuticals. By harnessing modern analytic methods and relevant *in vivo* HTS procedures, we showed rapid unraveling of cone snail venom components that have the *in vivo* effects of paralysis and/or lethality. By demonstrating the feasibility of this analytical pipeline, we hope to guide studies on venom composition and their bioactive components, and further contribute to unraveling the pharmacopeia of venom components that alter physiology, understanding their evolution and preferred molecular targets.

## 3. Materials and Methods

### 3.1. Chemical, Biological Reagents and Venoms

A Milli-Q Plus System (Millipore, Amsterdam, The Netherlands) was used for purifying the water used in this study. Acetonitrile (ACN) of UPLC/MS grade was from Concord, NC, USA. Formic acid (FA) of MS grade was from Biosolve (Valkenswaard, The Netherlands). Zebrafish embryo medium (also called ‘egg water’) was made by dissolving 2.94 g of NaCl, 0.13 g of KCl, 0.49 g of CaCl_2_·2H_2_O, 0.81 g of MgSO_4_·7H_2_O, and 2 drops of methylene blue solution (1 g L^−1^). NaCl, KCl, CaCl_2_·2H_2_O and MgSO_4_·7H_2_O were of analytical grade and purchased from Merck (Kenilworth, UK). Methylene blue (reagent grade) was from Sigma-Aldrich, Darmstadt, Germany. Lyophilized cone snail venoms of the species *Conus marmoreus*, *Conus ebraeus*, and *Conus bandanus* were dissolved in Milli-Q water to a final concentration of 0.2 mg/mL for analytical separation, nanofractionation and post-column bioassaying with parallel mass spectrometry (MS) measurement. These venoms were milked and collected by the Richard Lewis laboratory at the University of Queensland, Australia.

### 3.2. Cone Snail Venom Fractionation and Mass Spectrometry

Separation of the cone snail venom peptides for nanofractionation and subsequent post-column bioassaying with parallel MS assessment was performed by liquid chromatography (LC) using a Shimadzu system coupled to a high-resolution nanofractionation module and to MS via a 1:9 post-column flow split. Of 0.2 mg/mL cone snail venom solutions, a 50 µL sample was injected by a Shimadzu SIL-20A autosampler (Shimadzu Co., Kyoto, Japan). The LC separation was controlled by Shimadzu LabSolutions DB/CS v6.81 for which gradient elution was performed by a binary Shimadzu LC-30AB pump (A and B) at a flow rate of 0.5 mL/min. Mobile phase A consisted of water-ACN-FA (98:2:0.1, *v*/*v*/*v*) and mobile phase B of water-ACN-FA (2:98:0.1, *v*/*v*/*v*). The gradient applied was 0% to 20% B (5 min), 20–40% B (25 min), 40–90% B (4 min), 90% B (5 min) 90% to 0% B (1 min), and 0% B (10 min) on a 100 × 4.6 mm ID analytical column packed with Xbridge BEH300 reversed-phase C18 material (3.5 µm). The column eluate was split 1:9 by a low-dead-volume flow splitter. The smaller flow part was directed after the split (0.05 mL/min) to a Bruker Maxis HD Mass Spectrometer (Bruker Daltonics, Bremen, Germany). Electrospray ionization (ESI) in positive mode was used, and the following parameters were set: source temperature 200 °C; capillary voltage 4500 V; dry gas flow 4.0 L/min; mass range 500–3000 *m*/*z* with a data-sampling time of 1 s. Peptide masses (in Da) were calculated using DataAnalysis 5.0 software (Bruker, Darmstadt, Germany). The larger eluate part after the flow split was fractionated (12 s/well) onto black 96-well plates (Greiner Bio-One, Alphen aan den Rijn, The Netherlands) in serpentine fashion by a FractioMate^TM^ FRM100 nanofractionation collector (VU, Amsterdam, The Netherlands) controlled by FractioMator software. The outer wells of the well plate were not used for fractionation, which meant that only the region of the columns from 2 to 11 and the rows from B to G in the 96-well plate were used for venom nanofractionation. Fractionation was only performed within the time frame that the cone snail toxins eluted. After nanofractionation, the plates were vacuum centrifuged overnight to dryness at room temperature by a Christ Rotational Vacuum Concentrator RVC 2-33 CD Plus (Salm en Kipp, Breukelen, The Netherlands) with a cooling trap operating at −80 °C. The plates were then stored at −80 °C until bioassaying.

### 3.3. Zebrafish Animal Care and Handling

The zebrafish strain ABTL was kept at 28 °C in tanks with day/night light cycles of 10 h dark alternated with 14 h light periods. The zebrafish were handled in compliance with The Netherlands animal care regulation and standard operating procedures and in accordance with the Wet op de dierproeven (Article 9) of Dutch Law (National) and the same law administered by the Bureau of Animal Experiment Licensing, Leiden University (Local). This local regulation serves as the implementation of Guidelines on the protection of experimental animals by the Council of Europe, Directive 2010/63/EU. Zebrafish eggs were harvested at two hours post-fertilization (hpf), followed by incubation at 28 °C in zebrafish embryo medium. The developing embryos were kept in an incubator at 28 °C. Zebrafish larvae of an age below 6 dpf are not considered experimental animals in the Netherlands and therefore exempt from approval by the animal welfare committee according to the Dutch Animal Testing Act (Wet op Dierproeven, WOD).

### 3.4. Zebrafish Lethality Toxicity Assay

Dried cone snail peptides in the wells of the well plates used for the nanofractionation procedure were reconstituted in 50 µL of zebrafish embryo medium and then further diluted in zebrafish embryo medium onto new well plates using five times serial dilutions (i.e., 1× (stock), 5× dilution, 25× dilution, and 125× dilution using zebrafish embryo medium). Zebrafish embryos were generated by natural pair-wise mating. Twenty pairs of adult zebrafish were set up for each mating, and, on average, 100–150 embryos per pair were generated. Embryos were maintained at 28 °C in zebrafish embryo medium. Embryos were cleaned (dead embryos were removed) and sorted by the developmental stage at 1–2 days post-fertilization (dpf). The 4 dpf developed zebrafish embryos were placed into wells containing zebrafish embryo medium (50 µL) and diluted cone snail peptides collected from the nanofractionation analytics (50 µL). The zebrafish embryos will absorb the venom via their gills. At 5 dpf, the zebrafish embryos were examined and scored. For acute toxicity screening on the 96-well plates, all outer wells were filled with zebrafish embryo medium (100 µL/well) and not used for bioassaying in order to minimize edge effects. In each of the other wells, 50 µL embryo medium was placed together with one 4 dpf dechorionated zebrafish embryo. Then, 50 µL of diluted cone snail peptide fraction was added to initiate toxicity testing. For each fraction tested in a serial dilution fashion, the aim was to find the highest concentration of that fraction at which none of the zebrafish died (each fraction was tested in 1×, 5×, 25×, and 125× fold dilution for this) and the lowest concentration at which all zebrafish died. Between these concentrations, the cone snail peptide(s) in that particular well showed lethal toxicity effects.

The toxicity assessment of the zebrafish embryos was recorded at 4 dpf directly after adding cone snail peptides and again at 5 dpf, 24 h later. The following criteria should be met for the embryo to be scored as ‘dead’: (i) tissue was opaque (milky-white) in appearance instead of transparent, (ii) the heart was not beating, and (iii) motionless (no locomotor activity). Thus, we could determine the empirical (i.e., as fractionated compound concentrations in each well are not known) LD_50_ values for all the fractions collected during the nanofractionation by the serial dilution experiments (1×, 5×, 25×, and 125×). Data on lethality was acquired, which, for each dilution tested, 1 zebrafish was exposed in triplicate, and the number of dead zebrafish was counted after the exposure time. For the data acquisition and collection, the number of dead fish at each exposed dilution concentration was recorded manually in an Excel file. For the processing of the empirical LD_50_ values of the nanofractionated cone snail venom toxins, an R statistics script running in the program Rstudio™ (Version 2024.09.x) was applied to calculate the LD_50_ values (with standard deviation) of each venom fraction (the script is provided as a Supporting Information document “SI R Markdown file LD50s calculations”). The script applies probit regression to determine lethal doses based on zebrafish embryo mortality data. The LD_50_ values and standard deviations for each fraction were then plotted into chromatographic representation graphs, for which the X-axis represents retention time of fractionation, and the Y-axis represents the LD_50_ values calculated (with standard deviation; exported from the R script) of each fraction in logarithmic scale (unit set as E^0^, E^−1^, E^−2^, E^−3^). This way of performing statistical analysis using probit analysis enabled the extrapolation of the level of lethality of each fraction containing fractionated cone snail peptides.

### 3.5. Zebrafish Paralysis Assay

In each well of a 96-well plate used for nanofractionation, 200 µL zebrafish embryo medium was added per well and from there after allowing cone snail peptides in the wells to dissolve for 10 min, 50 µL was transferred to a well in a 48-well microtiter plate used for the zebrafish paralyzing assay, which already contained 150 µL zebrafish embryo medium and one 5 dpf dechorionated zebrafish embryo (at 28 °C). Wells A1 to F3 and A5 to F7 were used for testing fractions, and wells A4 to F4 and A8 to F8 were used for controls. Each fraction was tested at each dilution in triplicate. The 48-well plate was then placed into a water bath in the EthoVision™ system (Noldus, Wageningen, The Netherlands) at 28 °C for bioassaying at a duration of 50 min, of which 10 min was used for acclimatization and 40 min for experimental recording. During the experimental recording, the light and dark tests were carried out promptly (0–10 min light phase; 10–15 min dark phase; 15–25 min light phase; 25–30 min dark phase; and 30–40 min light phase). The movement of the embryos was filmed using the EthoVision™ system using an infrared camera. The video was recorded at 60 frames per second, which is sufficient to resolve the direction, speed, and duration of slow and/or spontaneous movement of the zebrafish embryos. The light and dark cycles introduced to the zebrafish embryos will result in stress-induced rapid movement behavior during the dark phases compared to the light phases, unless paralysis caused by one or more cone snail peptides had occurred. Under normal conditions, the movement of the fish will gradually decline over the time of observation due to fatigue and/or adaptation to the environment. For the data processing, the exported numerical data of the motion of the zebrafish was plotted in the following manner:

The movement ratio between each tested well and control well = ∑ ((the accumulated movement in two dark phases (10–15 min and 25–30 min) of each well containing nanofractionated toxins))/X (control).

X (control) = ∑ ((the accumulated movement in two dark phases (10–15 min and 25–30 min) of seven control wells))/7

Next, using this data, parallel chromatograms were plotted with the chromatographic retention time (in minutes) on the X-axis and the average movement ratio on the Y-axis, including standard deviations (these chromatographic plots were made in GraphPad Prism 9).

## Data Availability

The original contributions presented in this study are included in the article and Supplementary Material. Further inquiries can be directed to the corresponding author.

## Supplementary Materials

The following supporting information can be downloaded at: https://www.mdpi.com/article/10.3390/toxins17100504/s1, SI document: SI_Conoserver_org_search_results.xlsx [15,16,17,18,19,20,21,22,23,24,25,26,27,28,29,30,31,32,33,34,35,36,37,38,39,40,41,42,43,44,45,46,47,48,49,50,51,52,53,54,55,56,57,58,59].
